# Parvovirus B-19 Infection During Pregnancy

**DOI:** 10.1080/10647440300025518

**Published:** 2003

**Authors:** Anthony Al-Khan, Andrew Caligiuri, Joseph Apuzzio

**Affiliations:** Division of Maternal Fetal Medicine Department of Obstetrics Gynecology and Women’s Health, New Jersey Medical School 185 South Orange Avenue, MSB-E606 Newark NJ 07101-1709 USA

## Abstract

The development of an acute parvovirus B-19 infection during pregnancy can cause pregnancy complications ranging from early pregnancy loss to nonimmune hydrops. There is no treatment, but preventive measures can be used to decrease perinatal mortality. The diagnosis is made on the basis of clinical suspicion and serology. If the fetus exhibits hydrops in the latter part of pregnancy, the main treatment options include either correcting the associated anemia with intrauterine blood transfusion or birth with extrauterine management. Although the serious problems associated with this virus during pregnancy are uncommon, they can be fatal. In view of this, a pregnant woman who is antibody negative should try to avoid contact with large groups of young children in order to decrease contact with potential vectors.


*Infect Dis Obstet Gynecol 2003;11:175–179*

### Parvovirus B-19 infection during pregnancy

**Anthony Al-Khan, Andrew Caligiuri and Joseph Apuzzio**

*Division of Maternal Fetal Medicine, Department of Obstetrics, Gynecology and Women's Health, New Jersey Medical School, Newark, NJ*

The development of an acute parvovirus B-19 infection during pregnancy can cause pregnancy complications ranging from early pregnancy loss to nonimmune hydrops. There is no treatment, but preventive measures can be used to decrease perinatal mortality. The diagnosis is made on the basis of clinical suspicion and serology. If the fetus exhibits hydrops in the latter part of pregnancy, the main treatment options include either correcting the associated anemia with intrauterine blood transfusion or birth with extrauterine management. Although the serious problems associated with this virus during pregnancy are uncommon, they can be fatal. In view of this, a pregnant woman who is antibody negative should try to avoid contact with large groups of young children in order to decrease contact with potential vectors.

Key words: PERINATAL INFECTION, VIRUS, PREGNANCY

Parvovirus B-19 is an endemic viral infection that is most frequently seen among preschool and school-age children in the USA. B-19 has historically been referred to as 'slapped cheek' disease or the Fifth disease, because it was the fifth disease that was found to cause rash in children (the first four being measles, scarlet fever, rubella and Duke's disease; exanthem subitum is the sixth)[1]. Cossart *et al.*[2] discovered parvovirus B-19 in 1974. While screening for the hepatitis B infection, they were obtaining false-positive results. When these particles were studied, it was found that they were in fact a parvovirus. The specification as B-19 arose from the fact that the positive virus was found in the specimen that was on panel B and specimen 19. Soon afterwards, Anderson *et al.*[3] reported that parvovirus B-19 was the cause of Fifth disease.

Parvovirus B-19 is a linear, nonenveloped, encapsulated, icosahedral, single-stranded DNA virus with a terminal hairpin structure that forms a double-stranded section characteristic of the Parvoviridae family[4]. Parvovirus has been found in individuals of all ages, but the highest incidence of the virus is in patients aged 5 to 15 years.

### PATHOGENESIS

Parvovirus B-19 is the only parvovirus that is known to cause disease in humans. Another parvovirus, dependovirus, has been isolated in humans. However, it is not believed to cause illness, and is only thought to infect humans when it is associated with an adenovirus[5].

Cytological properties that are characteristic of parvovirus B-19 infection include giant pronormoblasts, cytoplasmic vacuolization, immature chromatin and large eosinophilic nuclear inclusion bodies[4]. The virus has a predilection for rapidly multiplying erythroid progenitor cells. This is explained by the presence of the virus receptor, globoside. Globoside, or blood group P antigen, is mainly found on the erythroid progenitor cells, erythroblasts and megakaryocytes. It is thought to be the cellular receptor for parvovirus B-19[6].

Correspondence to: Joseph Apuzzio, MD, Division of Maternal Fetal Medicine, Department of Obstetrics, Gynecology and Women's Health, New Jersey Medical School, 185 South Orange Avenue, MSB-E606, Newark, NJ 07101-1709, USA. Email: joseph.apuzzio@umdnj.edu

© 2003 The Parthenon Publishing Group 

Anderson *et al.*[3] experimentally exposed human subjects to parvovirus B-19 and studied its effects. They described what is now known as the classic biphasic parvovirus B-19 presentation. There is an average 6-day incubation period, after which the first phase of the disease begins with viremia, shedding of viral particles, erythroid-progenitor-cell depletion and parvovirus B-19-specific IgM antibodies. Virus particles can be found in nasal washes and gargles, but not in the urine. This is the period of transmissibility, and it is usually asymptomatic. However, patients may experience a nonspecific prodromal illness characterized by fever, malaise, myalgias, headache and pruritus. About 1 week into phase 1, the pediatric patient may present the classic 'slapped cheek' appearance. Phase 2 begins about 17 days after viral exposure, and is characterized by the appearance of parvovirus B-19-specific IgG antibodies and a corresponding decreasing viremia. The patient may experience joint stiffness that usually resolves within a few weeks[4]. Later, a lacey erythematous rash may develop on the trunk and extremities.

Although parvovirus B-19 is mainly considered to be a childhood disease, adults may also be infected. However, it is rare for adults to develop a rash. Instead, B-19 is better known for causing an aplastic crisis in adults with an underlying chronic anemia. This aplastic anemia is caused by the preference of B-19 for the rapidly multiplying erythroid progenitor cells[7].

Parvovirus B-19 can also cause persistent arthralgias. Studies have reported that arthropathy occurs in 60% of adults and less than 10% of children[8–10]. Most commonly the arthritis starts in the hands, knees, wrists and ankles. Most cases of B-19 arthropathy subside within 1 to 3 weeks. However, B-19 is probably an underdiagnosed cause of arthritis, as evidenced by a study in which 12% of patients in an arthritis clinic tested IgM positive[11]. This is much higher than the figure seen in the general population, and it suggests that a chronic parvovirus B-19 infection is the cause of many cases of arthritis.

Research has been undertaken to determine whether a correlation exists with regard to an increase in parvovirus B-19 in patients with blood transfusions. It was found that of 100 previously B-19-antibody-negative patients, 18 individuals seroconverted to antibody-positive within 3 months of the transfusion. Although this disease may not be very harmful to a healthy nonpregnant woman, it is of considerable significance to those who are pregnant. In the follow-up study, the seroconversion was reduced to zero with the use of additional screening for parvovirus and discarding of seropositive blood products[12].

### EFFECT OF PARVOVIRUS B-19 IN PREGNANCY

It has been noted that 3–19 % of pregnant women will serologically convert to IgM positive on exposure to parvovirus B-19, with a 33% vertical transmission rate[13]. Although the virus can be contracted in any trimester, the second trimester seems to carry the highest risk of fetal loss. It has also been noted, without a cause-and-effect relationship having been proven, that there is a 2–3% incidence of this virus in all spontaneous abortions[14].

Prospective studies of pregnant women have found that if the patient was susceptible to parvovirus B-19 infection, her fetuses might suffer from especially profound sequelae, such as fetal hydrops, anemia and even potential fetal demise. These studies did not find any increase in the incidence of congenital malformations due to parvovirus B-19[15]. A retrospective study of 300 babies with congenital anomalies found that the incidence of B-19 was no higher than in healthy infants. This has led us to believe that B-19 toxicity is embryologic rather than teratogenic[14].

The risk of fetal demise is highest in the first trimester, and is thought to be as high as 10% in women who are infected prior to 20 weeks' of gestation[16]. In a patient with a history of previous miscarriage due to parvovirus, the antibodies to B-19 will still be present during her next pregnancy, but there is no increase in the likelihood of miscarriage in the second pregnancy due to these antibodies[17]. B-19 is toxic not only to the maternal red-blood-cell precursors but also to fetal red-blood-cell precursors. Destruction of fetal red blood cells can lead to nonimmune hydrops fetalis. The risk of fetal hydrops is substantial, at around 3%[17].

The first case of fetal hydrops was seen in 1984, and since then many cases have been reported[18]. Hydrops can develop within 2 weeks, and may either resolve spontaneously or lead to fetal demise[19]. The severity of the fetal anemia is due to three factors, namely increased turnover of fetal red blood cells, rapidly expanding intravascular volume, and the inability of the immature immune system to fight the viral infection. The resulting decrease in fetal hemoglobin concentration and hematocrit due to the anemia can lead to high-output congestive heart failure causing fetal circulatory compromise. Red blood cells are not the only cell line that is affected by the virus. Parvovirus B-19 has also been known to infect megakaryocytes, placental cells, fetal liver cells and myocardial cells, but these cells are not thought to harbor any infectious virus or suffer permanent harm[20–23].

### CLINICAL FINDINGS OF ACTIVE DISEASE

The clinical findings are collectively referred to as erythema infectiosum. The different phases of the disease described earlier will have different findings on physical examination. The first phase of the disease will have nonspecific findings, including fever, malaise and other nonspecific viral symptoms. The patient may also present with a photosensitive erythematous rash on the face that spares the nasal and periorbital zones. This appearance gave rise to the term ‘slapped face’ syndrome. A second phase that is purely clinical, with no change in the immunoglobulins, can show a lacy, erythematous maculopapular exanthem on the trunk and extremities. The rash may itch, but if no itch is noted the diagnosis of parvovirus B-19 cannot be ruled out. The rashes will all usually resolve within 7 to 10 days.

In 60% of adults and 10% of children infected with parvovirus B-19, arthralgia/arthritis can be seen. Painful, swollen and stiff joints characterize this arthralgia. The most commonly affected joints are the wrist, hand, knee and ankle. This arthralgia usually lasts for 1 to 3 weeks, although rarely it can last for months to years[10–12].

### SCREENING AND DIAGNOSIS

The virus can usually be recognized by the clinical symptoms. In the adult this may sometimes be difficult, as other adult viruses may present very similarly. The diagnosis is made on the basis of clinical findings, and is confirmed by a serological test. An enzyme-linked immunosorbent assay (ELISA) can be undertaken to look for maternal antibodies[23].

Circulating IgM suggests the presence of an active infection, can be detected approximately 10 days after infection, and may remain positive for up to 6 months. IgG antibodies, which suggest a previous infection, are formed approximately 3 weeks after infection and may last for several years or even for life[24,25].

Maternal testing for parvovirus is not routinely performed for all pregnant women. To test a fetus for possible infection, a polymerase chain reaction (PCR) is performed on a sample of the amniotic fluid, as this test has a sensitivity greater than 97% and a specificity of 79–99%[28]. An additional option, namely obtaining a fetal cord blood sample, is not widely used because of the associated 1% fetal loss rate, and also because the IgM does not often appear in the fetal circulation until after 22 weeks’ gestation. Postpartum testing of the infant can be performed by a PCR on the bone marrow, but not by serology. This is because infants with congenital infection do not have virus particles circulating in the blood, and the virus can only be found in the bone marrow[27–29].

### MANAGEMENT/TREATMENT

Treatment of a parvovirus B-19 infection is dependent on the gestational age of the fetus. If the mother tests positive for IgM and negative for IgG (suggesting a new infection) at less than 20 weeks’ gestation, no treatment is necessary. This is because B-19 infection is associated with a very low risk of congenital anomalies[19]. These patients should undergo serial ultrasound examinations to monitor for the development of fetal hydrops. It is suggested that these ultrasound examinations be performed at weekly intervals, but there is some controversy over the necessity for repeated ultrasounds[19]. The fetal death rate is highest if infection occurs before 20 weeks' gestation, reaching approximately 10%, and it declines rapidly as the fetus ages[30]. Routine prenatal screening for parvovirus B19 is not advised. However, a woman may be tested if she develops symptoms suggestive of B-19 with a positive contact history.

If the fetus shows evidence of hydrops, the main treatment option is intrauterine blood transfusion to correct the associated anemia[19]. Testing of the middle cerebral artery is the most sensitive, noninvasive test for intrauterine anemia[31]. This test can be used as a diagnostic and screening tool to monitor intrauterine (hemolysis/anemia) insult[32]. If severe intrauterine hydrops/anemia is diagnosed at a gestational age of more than 28 weeks, delivery and extrauterine treatment and management should be considered. If the diagnosis is made at a gestational age of less than 28 weeks, an intrauterine blood transfusion should be considered, either intra-abdominally or by percutaneous umbilical blood sampling (PUBS)[16,17]. This treatment is also controversial, as it has been shown in some case reports that hydrops may resolve spontaneously without intervention[33,34]. Therefore careful patient selection and counseling should be performed prior to administering aggressive intrauterine therapy.

Termination of pregnancy is *not* indicated because there is no increase in the incidence of congenital malformations associated with acute infection[19,35,36].

If the pregnant woman tests positive for parvovirus B-19, she should be informed of the possible outcomes. During epidemic periods in specific schools, infection rates may be 5–20-fold higher, so avoiding potential infectious contacts, serological testing, or both may occasionally be appropriate[34,37,38]. Obstetricians may tailor their recommendations to individual patients.

### REFERENCES

1. Shapiro L. The numbered diseases: first through sixth. *J Am Med Assoc* 1965;194:210
2. Cossart YE, Field AM, Cant B, *et al.* Parvovirus-like particles in human sera. *Lancet* 1975;1: 72–3
3. Anderson MJ, Jones SE, Fisher-Jock SP, *et al.* Human parvovirus, the cause of erythema infectiosum (Fifth disease)? *Lancet* 1983;1:1378
4. Mandell GL, Bennett JE, Dolin R. *Mandell: Principles and Practice of Infectious Diseases*, 5th edn. New York: Churchill Livingstone, 2000:1685-7
5. Meyers C, Mane M, Kokorina N, *et al.* Ubiquitous human adeno-associated virus type 2 autonomously replicates in differentiating keratinocytes of a normal skin model. *Virology* 2000;272:338–46
6. Weigel-Kelley KA. Recombinant human parvovirus B19 vectors: erythrocyte P antigen is necessary but not sufficient for successful transduction of human hematopoietic cells. *J Virol* 2001;75:4110–16
7. Young N, Harrison M, Moore J, *et al.* Direct demonstration of the human parvovirus in erythroid progenitor cells infected *in vitro*. *J Clin Invest* 1989;84:1114–23
8. Agar EA, Chin TDY, Poland JD. Epidemic erythema infectiosum. *N Engl J Med* 1966;275: 1326–31
9. Woolf AD, Campion GV, Chishick A, *et al.* Clinical manifestations of human parvovirus B19 in adults. *Arch Intern Med* 1989;149:1153–6
10. Joseph PR. Fifth disease: the frequency of joint involvement in adults. *NY State J Med* 1986; 86:560–3
11. Klouda PT, Corbin SA, Bradley BA, *et al.* HLA and acute arthritis following human parvovirus infection. *Tissue Antigens* 1986;28:318–19
12. OBGYN.net. *New Process Screens Plasma for Human Parvovirus B19*, 2000: http://www.obgyn.net/newsrx/womens_health-blood_safety-20001225-1.asp
13. Public Health Laboratory Service Working Party on Fifth Disease. Prospective study of human parvovirus B19 infection in pregnancy. *Br Med J* 1990;30:1166–70
14. Van Elsacker-Niele AM, Salimans MM, Weiand HT, *et al.* Fetal pathology in human parvovirus B19 infection. *Br J Obstet Gynaecol* 1989;96:768–75
15. Rodis JF, Quinn DL, Gary GW Jr, *et al.* Management and outcomes of pregnancies complicated by human B19 parvovirus infection: a prospective study. *Am J Obstet Gynecol* 1990; 163:1168–71

16. Markenson GR, Yancey ML. Parvovirus B19 infections in pregnancy. *Semin Perinatol* 1998; 22:309
17. Brown KE, Green S, Antunez-de-Mayolo J. Congenital anemia following transplacental B19 parvovirus infection. *Lancet* 1994;343:895
18. Brown T, Anand A, Ritchie LD, *et al*. Intrauterine parvovirus infection associated with hydrops fetalis. *Lancet* 1984;2:1033–4
19. Fairley C, Smoliniec J, Caul O, *et al*. Observational study of the effect of intrauterine transfusions on outcome of fetal hydrops after parvovirus B19 infection. *Lancet* 1995:346:1335
20. Young NS, Mortimer PP, Moore JG, *et al*. Characterization of a virus that causes transient aplastic crisis. *J Clin Invest* 1984;73:224–30
21. Takahashi Y, Murai C, Shibata S, *et al*. Human parvovirus B19 as a causative agent for rheumatoid arthritis. *Proc Natl Acad Sci USA* 1998;95:8227–32
22. Karetnyi YV, Beck PR, Markin RS, *et al*. Human parvovirus B19 infection in acute fulminant liver failure. *Arch Virol* 1999;144:1713–24
23. Saal JG, Stendle M, Einsele H, *et al*. Persistence of B19 parvovirus in synovial membranes of patients with rheumatoid arthritis. *Rheumatology* 1992;12: 147–51
24. Schwarz TF, Jager G, Gilch S. Comparison of seven commercial tests for the detection of parvovirus B19-specific IgM. *Zentralbl Bakteriol* 1997;285:525
25. Torok T. Human parvovirus B19. In Remington J, Klein J, eds. *Infectious Diseases of the Fetus and Newborn Infant*. Philadelphia, PA: Saunders, 1995: 668–73
26. Sloots T. Evaluation of four commercial enzyme immunoassays for detection of immunoglobulin M antibodies to human parvovirus B19. *Eur J Clin Microbiol Infect Dis* 1996;15:758–61
27. Yamakawa Y, Oka H, Hori S, *et al*. Detection of human parvovirus by nested polymerase chain reaction. *Obstet Gynecol* 1995;86:126–9
28. Miller E, Jairley CK, Cohen BJ, *et al*. Immediate and long-term outcome of human parvovirus B19 in pregnancy. *Br J Obstet Gynaecol* 1998;105:174–8
29. Bahado-Singh RO. Middle cerebral artery Doppler velocimetric deceleration angle as a predictor of fetal anemia in Rh-allo immunized fetuses without hydrops. *Am J Obstet Gynecol* 2000;183:746–51
30. Sheikh AU, Ernest JM, O'Shea M. Long-term outcome in fetal hydrops from parvovirus B19 infection. *Am J Obstet Gynecol* 1992;167:461–6
31. Cosmi E, Mari G, Delle Chiaie L, *et al*. Non-invasive diagnosis by Doppler ultrasonography of fetal anemia resulting from parvovirus infection. *Am J Obstet Gynecol* 2002;187:1290–3
32. Humphrey W, Magoon M, O'Shaughnessy T. Severe non-immune hydrops secondary to parvovirus B19 infection: spontaneous reversal *in-utero* and survival of a term infant. *Obstet Gynecol* 1991;78:900–2
33. Pryde PG, Nugent CE, Pridjian G, *et al*. Spontaneous resolution of nonimmune hydrops fetalis secondary to human parvovirus B19 infection. *Obstet Gynecol* 1992;79:859
34. Adler SP. Risk of human parvovirus B19 infections among school and hospital employees during endemic periods. *J Infect Dis* 1993;168:361–8
35. Rotbart HA. Human parvovirus infections. *Annu Rev Med* 1990;41:25–34
36. Eis-Hübinger AM, Dieck D, Schild R, *et al*. Parvovirus B19 infection in pregnancy. *Intervirology* 1998;41:178–84
37. Torok TS, Wang QY, Gary GW, *et al*. Prenatal diagnosis of intrauterine infection with parvovirus B19 by the polymerase chain reaction technique. *Clin Infect Dis* 1992;14:149–55
38. Clewley JP. Polymerase chain reaction assay of parvovirus B19 DNA in clinical specimens. *J Clin Microbiol* 1989;27:2647–51

RECEIVED 01/27/03; ACCEPTED 05/16/03
